# Sex-Stratified Prediction Models for 5-Year Nonalcoholic Fatty Liver Disease Risk in Thyroid Cancer Patients: A Nationwide Cohort Study

**DOI:** 10.3390/biomedicines13092250

**Published:** 2025-09-12

**Authors:** Young Bin Cho, Kyoung Sik Park

**Affiliations:** 1 Department of Medicine, Graduate School of Konkuk University, Seoul 05029, Republic of Korea; cyb6602@konkuk.ac.kr; 2 Department of Surgery, Konkuk University School of Medicine, Seoul 05030, Republic of Korea; 3 Department of Surgery, Konkuk University Medical Center, Seoul 05030, Republic of Korea

**Keywords:** thyroid cancer, nonalcoholic fatty liver disease, prediction model, nationwide cohort

## Abstract

**Background/Objectives**: Nonalcoholic fatty liver disease (NAFLD) is a significant complication among survivors of thyroid cancer; however, existing prediction models for NAFLD remain inadequate. Our objective was to develop survival prediction models for 5-year risk of NAFLD in patients diagnosed with thyroid cancer. **Methods**: Utilizing the Korean National Health Insurance Service claims database, we selected 3644 post-thyroidectomy patients with thyroid cancer between 2004 and 2014. Following a 7:3 stratified division into training and test datasets, we developed sex-stratified survival models using random survival forest (RSF) and Cox proportional hazards regression (Cox). The evaluation of prediction models was performed using Harrell’s concordance index (C-index), time-dependent area under the curve (AUC), and risk stratification analysis. **Results**: In the female cohort, the Cox model exhibited a superior C-index of 0.67 (95% CI 0.61–0.72), surpassing the RSF model, which had a C-index of 0.62 (95% CI 0.57–0.68). Notably, age-stratified Cox models for females demonstrated enhanced performance compared to the unstratified female Cox model. Conversely, male-specific models did not show significant performance in NAFLD. Risk stratification analysis revealed that the female-specific models effectively categorized patients into low- and high-risk groups, with statistical significance (*p* < 0.001). **Conclusions**: This study constructed well-performing time-to-event prediction models for NAFLD of female patients with thyroid cancer, which is significant in risk stratification.

## 1. Introduction

Thyroid cancer is a prevalent endocrine malignancy, and its incidence has progressively increased over the past few decades [1,2]. In South Korea, it is the most frequently diagnosed cancer among middle-aged individuals, with a 5-year relative survival rate of 100% [3]. Nevertheless, individuals diagnosed with thyroid cancer are at an increased risk of developing metabolic disorders, such as liver disease, cardiovascular disease, and osteoporosis [4,5,6]. Considering the high survival rates and predominantly younger demographics of patients with thyroid cancer, it is crucial to implement effective strategies to mitigate complications and enhance their overall quality of life.

Nonalcoholic fatty liver disease (NAFLD) is a metabolic disorder characterized by lipid accumulation, primarily triglycerides, within hepatocytes. Previous studies have demonstrated that NAFLD not only increases the risk of liver-related disorders, liver transplantation, and mortality, but is also closely linked to conditions such as metabolic syndrome and atherosclerotic cardiovascular disease [7,8,9].

Thyroid cancer survivors may have an increased susceptibility to NAFLD, primarily due to disruption of thyroid hormone homeostasis that occurs during treatment and long-term management. Thyroid hormones play a crucial role in regulating hepatic lipid metabolism, affecting processes such as fatty acid β-oxidation, de novo lipogenesis, and cholesterol balance. Even minor deviations in thyroid axis function have been linked to hepatic steatosis and fibrosis [10,11]. Fluctuations in thyroid hormone levels can lead to a reduction in mitochondrial oxidative capacity and impair lipid metabolism while concurrently activating transcriptional programs such as SREBP-1c and ChREBP, which promote de novo lipogenesis. Concurrently, elevated TSH levels and altered deiodinase activity may increase hepatic lipid influx and reduce intrahepatic T3 action, resulting in an intrahepatic hypothyroid condition that promotes triglyceride accumulation and fibrosis. Collectively, these mechanisms offer a biological explanation for the increased susceptibility of thyroid cancer survivors to NAFLD [11]. A nationwide cohort study has shown that Korean patients with thyroid cancer have more than a two-fold increased risk of developing NAFLD compared with matched controls, even after adjusting for metabolic comorbidities [4]. Similarly, the Rotterdam Study reported that lower thyroid function, characterized by higher TSH and lower FT4 levels, was independently associated with an increased incidence of NAFLD and hepatic fibrosis [12]. Therefore, it is imperative to predict and manage the risk of NAFLD in patients with thyroid cancer.

Prediction models quantify the probability of outcomes for individual patients, thereby offering concrete evidence to support clinical decision-making during routine care. These risk estimates are used for the early identification of high-risk groups, tailoring follow-up screening intervals, and optimizing resource allocation. For example, FRAX calculates the 10-year fracture risk to suggest thresholds for bone mineral density testing or treatment initiation [13]. Similarly, QRISK3 is widely employed to determine the necessity of preventive therapy based on a patient’s 10-year risk of cardiovascular disease [14]. Consequently, it is imperative to identify the relevant predictors and develop a predictive model for NAFLD risk in thyroid cancer survivors. However, research in this field is limited. Although several studies have examined the association between thyroid cancer and NAFLD, they have largely focused on general epidemiological associations rather than developing clinically applicable prediction tools. Furthermore, the effect size of the association between thyroid cancer and NAFLD risk varies significantly according to sex [4]. However, previous studies have rarely incorporated sex-specific metabolic differences that may influence NAFLD risk. This underscores the need to develop sex-stratified prediction models for NAFLD in patients with thyroid cancer.

Therefore, we aimed to develop and internally evaluate sex-stratified time-to-event prediction models to estimate the 5-year risk of NAFLD among thyroid cancer survivors. We compared the Cox proportional hazards and random survival forest approaches and prespecified an age-stratified analysis among women.

## 2. Materials and Methods

### 2.1. Data Source

This study utilized the National Health Insurance Service (NHIS)-Sample Cohort of South Korea, encompassing 1,134,108 individual qualifications, medical diagnoses (in accordance with the International Classification of Disease, Tenth Revision [ICD-10]), health checkups, and prescriptions from 1 January 2002 to 31 December 2019.

### 2.2. Study Design & Population

**Figure 1:**
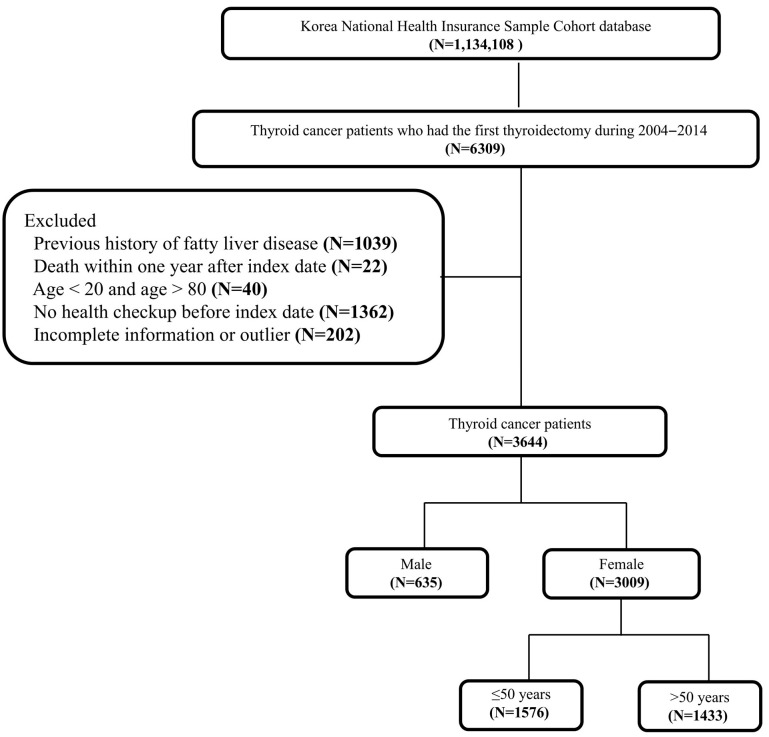
Flowchart of study population. The index date was the date on which the first date of thyroidectomy.

Flow charts of the study population and the study design are presented in Figure 1 and Supplementary Figure S1. We selected patients with thyroid cancer (C73) who underwent their first thyroidectomy between 1 January 2004 and 31 December 2014. Subsequently, we applied the following exclusion criteria: (1) history of fatty liver disease; (2) death within 1 year after the index date; (3) age under 20 years or over 80 years; (4) no health checkup before the index date; (5) incomplete information or outliers.

Each cohort was partitioned into training and test sets through stratified splitting at a 7:3 ratio, ensuring preservation of the distribution of event occurrences [15]. The primary outcome was the 5-year incidence of NAFLD (K760, K758) from the index date. The index date was defined as the date of the first thyroidectomy. Definitions of the variables and outcomes are presented in Supplemental Table S1.

### 2.3. Model Construction

We developed and compared two time-to-event prediction models, RSF and Cox. RSF is a machine learning method that builds an ensemble of decision trees in survival data [16], whereas Cox is a traditional regression-based survival model [17]. Model development and feature selection were conducted exclusively on the training set. All continuous variables were standardized to z-scores using the distribution of the training and test sets to prevent scale-dependent penalty bias. Feature selection was performed using the Boruta algorithm, and only variables classified as “confirmed” were selected. For the RSF model, hyperparameter tuning was conducted using a grid search strategy, exploring mtry (4, 5, 6, 7, 8, 9, 10), ntree (30, 50, 100, 300, 500), and node size (3, 5, 10, 15) within the training dataset. To evaluate each combination, a five-fold cross-validation approach was employed, and the hyperparameter set that yielded the highest mean concordance index (C-index) was selected (Supplementary Table S2). Finally, both the RSF and Cox models were developed using five-fold cross-validation.

### 2.4. Model Evaluation

The model performance was assessed using both the training and test sets. We evaluated the overall discriminative ability using the C-index. A C-index of 0.5 indicates a performance equivalent to random guessing, whereas a C-index of 1.0 reflects a perfectly accurate prediction [18]. In addition, we used the time-dependent area under the ROC curve (AUC), which is a survival analysis metric that evaluates a model’s discriminative ability at specific time points, accounting for censored data and varying event risk over time [19]. The time-dependent AUC was assessed at 1-year intervals from the index date to over 5 years.

### 2.5. Risk Stratification

The two models independently calculated the risk scores for each patient. To establish the cutoff point for the risk score, we employed maximally selected log-rank statistics using the maxstat package. The optimal cutoff points identified within the training set delineated the low-risk (risk score ≤ cutoff point) and high-risk (risk score > cutoff point) group. This method systematically evaluates all possible cutoff values of the continuous risk score and selects the point that maximizes group separation based on survival differences, as measured by the log-rank statistic [20]. Therefore, it identified the threshold that best discriminates between patients with a higher and lower incidence of NAFLD within the follow-up period.

### 2.6. Statistical Analysis

The baseline characteristics of the study population were assessed using the Wilcoxon test for continuous variables, whereas categorical variables were analyzed using the χ^2^ test or Fisher’s exact test. For risk stratification, the Kaplan–Meier method was employed to estimate the cumulative incidence of NAFLD in both high- and low-risk groups, and the log-rank test was used to compare the incidence curves. Data preprocessing was conducted using the SAS software (version 9.4; SAS Institute Inc., Cary, NC, USA). Statistical analyses, model construction, and model evaluation were performed using R, version 4.3.2 (R Project for Statistical Computing).

## 3. Results

### 3.1. Baseline Characteristics

The study included 3644 patients, comprising 635 males and 3009 females, with events occurring in approximately 10% of each group, as detailed in Table 1. Female participants were further categorized into subgroups based on whether they were below or above 50 years of age at the index date, as this age typically marks the onset of menopause in females (Supplementary Table S3). Among females aged 50 years or younger (younger females), 1576 patients had a 7.9% incidence of NAFLD. In contrast, among females over 50 years of age (older females), there were 1433 patients with a 12.8% incidence of NAFLD.

Across all cohorts, including males, overall females, younger females, and older females, no statistically significant differences were observed between the training and test sets in terms of baseline characteristics (Supplementary Tables S4–S7).

### 3.2. Model Performance

**Figure 2:**
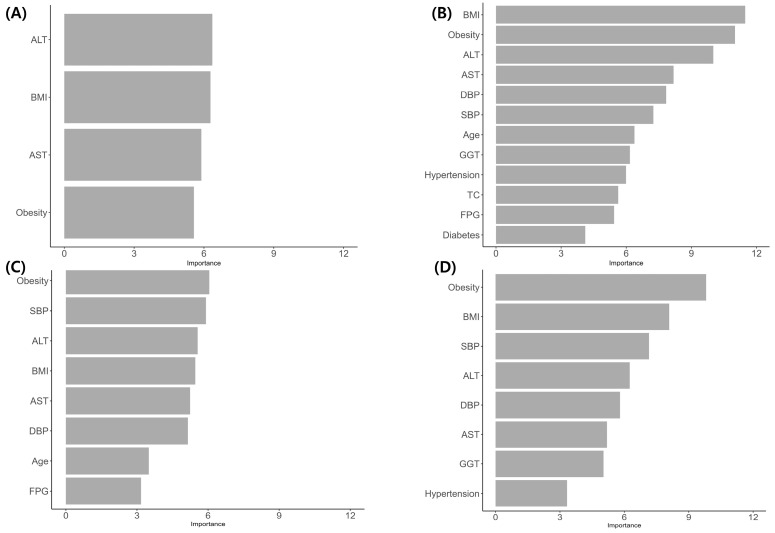
Importance of feature selection. (**A**) male (**B**) all female (**C**) younger (≤50 years) female (**D**) older (>50 years) female. ALT, alanine aminotransferase; AST, aspartate aminotransferase; BMI, body mass index; DBP, diastolic blood pressure; SBP, systolic blood pressure; GGT, gamma-glutamyl transferase; TC, total cholesterol; FPG, fasting plasma glucose.

All variables were used for feature selection, underscoring the importance of potential variables in predicting the risk of NAFLD (Figure 2).

In the male cohort, the RSF model attained a C-index of 0.59 (*p* = 0.046), indicating a level of discrimination akin to randomness. In contrast, the Cox model achieved a marginally higher C-index of 0.64, although this did not reach statistical significance (*p* = 0.541). These findings suggest that male-specific models have limited prognostic utility.

**Figure 3:**
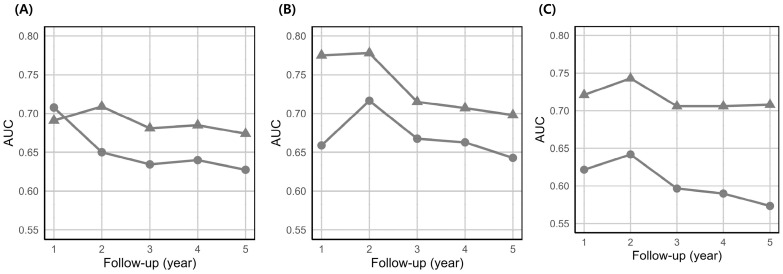
Time-dependent AUC of female-specific models in test set. (**A**) all female (**B**) younger (≤50 years) female (**C**) older (>50 years) female. ▲, Cox proportional hazards regression (Cox) model. ●, Random survival forest (RSF) model. AUC, area under the curve.

Across all female cohorts, the Cox model demonstrated a higher C-index than the RSF model (Table 2). Notably, constructing separate models for younger and older females slightly enhanced discrimination relative to the model developed for the entire female population. Furthermore, the Cox models consistently exhibited higher time-dependent AUC values than the RSF models (Figure 3).

Time-dependent AUC values for the male cohort are shown in Supplemental Figure S2. All results pertaining to the model performance in the training set are depicted in Supplemental Figure S3 and Supplementary Table S8.

### 3.3. Risk Stratification

**Figure 4:**
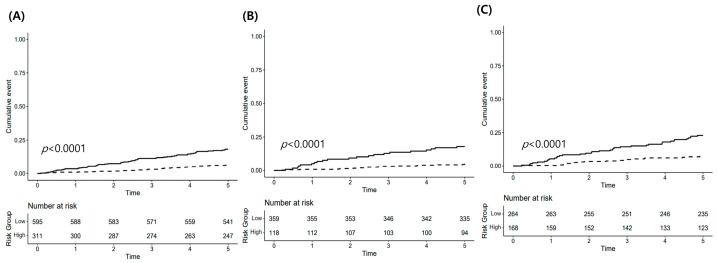
Risk stratification of female-specific Cox models in test set. (**A**) all female (**B**) younger (≤50 years) female (**C**) older (>50 years) female. Solid line represents the high-risk group. Dash line represents the low-risk group.

All female-specific Cox models demonstrated a statistically significant distinction between the high- and low-risk groups (*p* < 0.001), with the hazard ratio indicating that the high-risk group experienced a substantially greater number of events (Figure 4, Table 3). Consequently, the risk stratification of the Cox models effectively differentiated the potential risk of NAFLD between the low- and high-risk groups among all female patients with thyroid cancer. However, the RSF models only significantly stratified the risk group within the younger female cohort (Supplementary Figure S4 and Supplementary Table S9).

Male-specific models did not exhibit statistically significant risk stratification between the high- and low-risk groups in the test set (Supplementary Figure S5, Supplementary Table S10). The results of risk stratification in the training set are presented in Supplementary Figures S6–S8 and Supplementary Tables S11–S13.

## 4. Discussion

This study presents the development and assessment of sex-stratified time-to-event prediction models for estimating the 5-year risk of NAFLD in patients with thyroid cancer. The constructed Cox model exhibited superior performance compared to the RSF model in the female cohort. Although the RSF model demonstrated a higher C-index and AUC across all time points in the training set, it appeared to be overfitted. The limited number of NAFLD events and the use of feature selection methods may still pose a risk of overfitting, although we implemented a stratified training–test split and five-fold cross-validation. To address overfitting, independent cohorts are needed to validate the robustness of our models using resampling-based approaches.

Conversely, male-specific models were poorly evaluated, as evidenced by low performance metrics or a lack of statistical significance. This indicates that the NHIS dataset lacks adequate predictors for precise NAFLD risk estimation in male patients with thyroid cancer. Notably, feature selection identified eight or 12 predictors in the female cohort, whereas only four were identified in the male cohort. This likely reflects both the lower number of NAFLD events and the insufficient diversity of available predictors in the male subgroup. Similar sex-specific differences have been reported in a previous study, where the association between NAFLD and diabetes or hypertension was more significant in females than in males [21]. This suggests that the currently available variables may capture female risk profiles more effectively. Therefore, we acknowledge the low performance of male-specific models as a negative finding that warrants cautious interpretation. Future models for men should incorporate additional features, such as visceral adiposity or sex hormone levels, and should be developed in larger or hospital-based cohorts.

This study stratified female participants into two age groups, younger (≤50 years) and older (>50 years), acknowledging the significant physiological changes associated with menopause. The analysis revealed that two age-stratified Cox models for females demonstrated superior performance compared to an unstratified female Cox model. This is consistent with a recent meta-analysis of 12 cross-sectional studies. This meta-analysis reported that postmenopausal women have approximately 2.4-fold higher odds of NAFLD than premenopausal women (95% CI, 1.99–2.82) [22]. This supports our stratification of female participants by the menopausal proxy, highlighting the distinct metabolic milieu and NAFLD susceptibility before and after menopause.

The risk score was calculated for each patient, categorizing them into high- and low-risk groups. This enabled the reliable stratification of the 5-year risk of NAFLD. Identifying high-risk groups and accurately predicting the long-term risk of NAFLD in patients with thyroid cancer is clinically significant. The risk score derived from female-specific Cox models effectively stratified NAFLD risk in patients with thyroid cancer. Using the constructed Cox models, clinicians can assess the potential NAFLD risk in female patients and provide tailored recommendations for liver screening and personalized treatment regimens.

Furthermore, the Boruta algorithm identified BMI, ALT, AST, and obesity as significant risk variables across all populations, irrespective of sex and age. This finding aligns with the prognostic factors identified in previous studies [23,24,25]. Prior research has suggested a dose-dependent association between BMI and NAFLD risk and indicated that obese patients have an elevated risk of developing NAFLD [23]. The ALT/AST ratio is a significant marker of NAFLD risk [25]. Moreover, predictive models of NAFLD in healthy individuals have consistently incorporated similar variables, such as ALT, triglycerides, waist circumference, and metabolic score for insulin resistance, as strong predictors of NAFLD [26]. Our identification of BMI, ALT, AST, and obesity as the top predictors aligns with these findings. In female patients with thyroid cancer, blood pressure was consistently identified as a significant factor in both the overall and age-stratified female cohorts [27,28]. This is consistent with previous research suggesting a relationship between blood pressure categories and NAFLD risk.

Female-specific models, particularly two age-stratified Cox models, may serve as clinically effective strategies for NAFLD surveillance, facilitating the early identification of high-risk individuals and the efficient allocation of diagnostic resources. These models can be incorporated into new, easily accessible devices to support the diagnosis and timely management of patients with diabetes. To apply our prediction models in practice, we will employ a two-step screening workflow, which comprises our female-specific Cox models as the initial triage tool, followed by hepatic assessment exclusively for model-defined high-risk patients, utilizing a stepped-wedge cluster randomized design. Previous studies have suggested that liver disease surveillance could benefit from stepped-wedge or cluster-randomized frameworks to enhance the efficiency of liver care [29].

To facilitate implementation, we will develop an interactive web-based application that utilizes Shiny [30]. Clinicians will input individual patient predictor values to obtain time-to-event outputs, including cumulative NAFLD risk at specific time points as well as the overall 5-year risk, expressed on a scale from 0 to 1. Additionally, they will receive a categorical risk label with color coding, accompanied by automated recommendations for ordering hepatic imaging or liver biopsy, assessing ALT levels, initiating lifestyle modification counseling, and referring high-risk patients to hepatology. Concurrently, we will package the Cox model as an FHIR-compliant RESTful API to enable seamless integration within electronic medical records, thereby allowing for automatic computation of NAFLD risk [31]. If implemented, the clinical impact of API will be prospectively evaluated in a separate study.

We intend to conduct a stepped-wedge trial involving approximately 1000 thyroid cancer survivors to prospectively evaluate the impact of the model on automated recommendations. By utilizing a risk score derived from specific variables, clinicians can classify patients as either low- or high-risk for NAFLD. This will be facilitated through a clinician’s decision-support interface targeting high-risk groups [32]. This approach aims to validate the predictive accuracy and demonstrate its potential to significantly enhance patient health outcomes and workflow efficiency in real-world settings.

This study acknowledges several limitations. First, the research utilized data from the National Health Insurance Service claims, which lacks certain variables such as menopausal status, TSH, histological subtype of the tumor, bilirubin, and ammonia, all of which could serve as critical predictors for NAFLD risk. Although TSH is a key biomarker of metabolic changes after thyroid cancer, our primary aim was to estimate the risk of NAFLD from the index date, defined as the first date of thyroidectomy, without incorporating post-treatment variables such as TSH levels. By doing so, we intended to provide a pragmatic prediction tool that can be applied at baseline using only routinely available health examination data and hospital utilization records without requiring specialized tests. On the other hand, to address the absence of menopausal status, we stratified female participants into younger and older subgroups based on age 50 years and developed age-stratified prediction models. Within this scope, female-specific models yielded acceptable performance and demonstrated clinical utility for risk stratification. In contrast, the male-specific models did not achieve adequate predictive accuracy, underscoring the need for future research to incorporate thyroid-related biomarkers, such as TSH, as well as other relevant clinical variables to improve performance. Second, the models specific to male participants demonstrated a suboptimal performance. Therefore, we do not recommend their clinical application in male. Future studies should incorporate a broader range of predictors, such as visceral adiposity and sex hormones, and should be conducted in larger cohorts. Ultimately, developing a distinct predictive model for male patients using hospital-based datasets may provide more accurate risk estimations. Third, the study did not include an external validation cohort such as hospital-based data. Although five-fold cross-validation was conducted for the model development, all folds were derived from the same data source, which may limit the generalizability of our findings. Therefore, future studies should perform external validation using independent datasets and update the models before clinical implementation. We plan to deploy the prediction models as a RESTful API service that can be integrated into hospital EMR systems, thereby prospectively applying them to newly diagnosed thyroid cancer patients. Fourth, as our study was conducted exclusively on a Korean population, the generalizability of the model to other races or ethnic groups remains uncertain. Differences in genetic backgrounds, lifestyles, and metabolic risk profiles across populations may affect the applicability of our findings. Broader future studies are necessary to validate and refine the model using multiethnic cohorts. Fifth, our prediction models were not designed to elucidate causal relationships. Although prior studies have linked thyroid dysfunction to hepatic steatosis and fibrosis, establishing a causal relationship between alterations in thyroid function and incident NAFLD remains challenging [10,11,12]. Moreover, while previous work has examined thyroid function changes and potential hepatic fat accumulation, the actual clinical effects may differ in vivo. Consequently, additional research is required to clarify the biological basis of the relationship between thyroid function and fatty liver disease. Finally, we acknowledge the recent shift to the concepts of metabolic dysfunction–associated fatty liver disease (MAFLD) and metabolic dysfunction–associated steatotic liver disease (MASLD), which highlight the role of metabolic dysfunction in steatotic liver diseases [33,34]. However, our study period ended in 2019, when NAFLD was the prevailing entity for case identification. Therefore, as our study defined the outcome as NAFLD based on the ICD-10, our findings should be interpreted in that context. Future studies applying contemporary MAFLD and MASLD criteria are needed to confirm and extend these results.

Our study had several strengths. First, it represents an inaugural effort to develop predictive models for NAFLD in patients with thyroid cancer using a nationwide cohort. These models have the potential to provide clinicians with valuable guidance in clinical decision-making, thereby facilitating the management of complications in individual patients. Second, our model can predict the 5-year risk of NAFLD at the time of thyroid cancer diagnosis without necessitating additional tests, as it relies solely on routinely collected variables from claims data. This approach is cost-effective and enhances patient convenience by eliminating unnecessary procedures. Finally, we developed time-to-event prediction models that retained information from right-censored observations throughout the follow-up period and generated individual risk curves at one-year intervals over five years. This capability enables personalized screening schedules and actionable clinical decision support, which single-time-point classification models cannot provide. This underscores the high translational value of our approach for direct integration into clinical practice.

## 5. Conclusions

This study developed four different models for NAFLD prediction in patients with thyroid cancer, accounting for biological sex and age. In female cohorts, the Cox model has better discrimination ability than the RSF model, and age-stratified Cox models have better discrimination ability than unstratified Cox models. However, male-specific prediction models showed no significant performance. Finally, we conducted risk stratification, which will help doctors identify high-risk patients for NAFLD.

## Figures and Tables

**Table 1:** Baseline characteristics of the study populations.

Variables	All (N = 3644)	Male (N = 635)	Female (N = 3009)	*p* Value
Age	50.0 (42.0–56.0)	47.0 (39.0–55.5)	50.0 (42.0–57.0)	<0.001
Income				<0.001
Low	931 (25.5)	80 (12.6)	851 (28.3)	
Middle	972 (26.7)	146 (23.0)	826 (27.5)	
High	1741 (47.8)	409 (64.4)	1332 (44.3)	
Residence				0.853
Urban	1827 (50.1)	321 (50.6)	1506 (50.0)	
Rural	1817 (49.9)	314 (49.4)	1503 (50.0)	
Disability	131 (3.6)	28 (4.4)	103 (3.4)	0.273
Insurance type				<0.001
Self-employed	978 (26.8)	113 (17.8)	865 (28.7)	
Work-employed	2666 (73.2)	522 (82.2)	2144 (71.3)	
Smoking status				<0.001
Never	3164 (86.8)	248 (39.1)	2916 (96.9)	
Ex	240 (6.6)	200 (31.5)	40 (1.3)	
Current	240 (6.6)	187 (29.4)	53 (1.8)	
Alcohol intake				<0.001
0	2755 (75.6)	268 (42.2)	2487 (82.7)	
1	715 (19.6)	256 (40.3)	459 (15.3)	
2	139 (3.8)	92 (14.5)	47 (1.6)	
≥3	35 (1.0)	19 (3.0)	16 (0.5)	
Alcohol binge	1342 (36.8)	353 (55.6)	989 (32.9)	<0.001
Regular exercise	490 (13.4)	83 (13.1)	407 (13.5)	0.809
Health examination				
BMI (kg/m^2^)	23.4 (21.5–25.6)	24.8 (23.1–26.7)	23.1 (21.2–25.3)	<0.001
BMI				<0.001
Underweight	128 (3.5)	9 (1.4)	119 (4.0)	
Normal	1455 (39.9)	145 (22.8)	1310 (43.5)	
Overweight	883 (24.2)	182 (28.7)	701 (23.3)	
Obese	1178 (32.3)	299 (47.1)	879 (29.2)	
SBP (mmHg)	120 (110–130)	124 (116–131)	120 (110–130)	<0.001
DBP (mmHg)	75 (70–80)	80 (70–85)	74 (69–80)	<0.001
FPG (mg/dL)	92 (85–100)	94 (87–105)	92 (85–100)	<0.001
TC (mg/dL)	191 (168–218)	192 (170–219)	191(168–218)	0.661
AST (IU/L)	21 (18–26)	24 (20–29)	21 (17–25)	<0.001
ALT (IU/L)	18 (14–26)	25 (19–35)	17 (13–24)	<0.001
GGT (IU/L)	18 (13–27)	32 (22–48)	17 (12–23)	<0.001
Comorbidities				
Dyslipidemia	1784 (49.0)	325 (51.2)	1459 (48.5)	0.234
Diabetes	949 (26.0)	174 (27.4)	775 (25.8)	0.419
Hypertension	1515 (41.6)	352 (55.4)	1163 (38.7)	<0.001
Obesity	1802 (49.5)	409 (64.4)	1393 (46.3)	<0.001
CCI				0.440
≤2	350 (55.1)	1605 (53.3)	1955 (53.6)	
>2	285 (44.9)	1404 (46.7)	1689 (46.4)	
Thyroidectomy type				0.737
Lobectomy	663 (18.2)	119 (18.7)	544 (18.1)	
Total thyroidectomy	2981 (81.8)	516 (81.3)	2465 (81.9)	
Outcome				
NAFLD	371 (10.2)	64 (10.1)	307 (10.2)	0.983

Values are expressed as mean (standard deviation) or number (%). BMI, body mass index; SBP, systolic blood pressure; DBP, diastolic blood pressure; FPG, fasting plasma glucose; TC, total cholesterol; ALT, alanine aminotransferase; AST, aspartate aminotransferase; GGT, gamma-glutamyl transferase; CCI, Charlson Comorbidity Score; NAFLD, nonalcoholic fatty liver disease.

**Table 2:** C-index of prediction models in test set.

	RSF		Cox	
	C-Index (95% CI)	*p* Value	C-Index (95% CI)	*p* Value
Male	0.59 (0.48–0.71)	0.046	0.64 (0.51–0.76)	0.541
Female	0.62 (0.57–0.68)	0.005	0.67 (0.61–0.72)	<0.001
≤50 years	0.64 (0.55–0.74)	0.011	0.69 (0.61–0.78)	0.004
>50 years	0.57 (0.50–0.64)	0.110	0.70 (0.64–0.76)	0.003

RSF, random survival forest; Cox, Cox proportional hazards regression; C-index, concordance index; CI, confidence interval.

**Table 3:** Hazard ratio of the female-specific Cox models in test set.

		Total	Event (%)	1000 PY	HR (95% CI)	*p* Value
All female	Low risk	595	37 (6.22)	4704.14	1.00	
	High risk	311	56 (18.01)	14,588.72	3.11 (2.05–4.71)	<0.001
≤50 years	Low risk	359	18 (5.01)	3764.73	1.00	
	High risk	118	21 (17.80)	14,496.59	3.84 (2.04–7.20)	<0.001
>50 years	Low risk	264	19 (7.20)	5477.56	1.00	
	High risk	168	38 (22.62)	18,942.98	3.47 (2.00–6.02)	<0.001

Cox, Cox proportional hazards regression; 1000 PY, per 1000 person-year; HR, hazard ratio; CI, confidence interval.

## Data Availability

Owing to the anonymization of the National Health Insurance Service data and the stringent control of researchers’ access, the requirement for informed consent was waived by the National Health Insurance Service.

## Supplementary Materials

The following supporting information can be downloaded at: https://www.mdpi.com/article/10.3390/biomedicines13092250/s1, Figure S1: Study design; Figure S2: Time-dependent AUC of male-specific models; Figure S3: Time-dependent AUC of female-specific models in training set; Figure S4: Risk stratification of female-specific RSF models in test set; Figure S5: Risk stratification of male-specific models in test set; Figure S6: Risk stratification of female-specific Cox models in training set; Figure S7: Risk stratification of female-specific RSF models in training set; Figure S8: Risk stratification of male-specific models in training set; Table S1: Definitions of variables and outcome; Table S2: Optimal hyperparameters of random survival forest; Table S3: Baseline characteristics of female populations; Table S4: Baseline characteristics of the training and test set in male cohort; Table S5: Baseline characteristics of the training and test set in female cohort; Table S6: Baseline characteristics of the training and test set in younger (≤50 years) female cohort; Table S7: Baseline characteristics of the training and test set in older (>50 years) female cohort; Table S8: C-index of prediction models in training set; Table S9: Hazard ratio of the female-specific RSF model in test set; Table S10: Hazard ratio of the male-specific models in test set; Table S11: Hazard ratio of the female-specific Cox model in training set; Table S12: Hazard ratio of the female-specific RSF model in training set; Table S13: Hazard ratio of the male-specific models in training set.

## Abbreviations

The following abbreviations are used in this manuscript:

NAFLD	Nonalcoholic fatty liver disease
RSF	Random survival forest
Cox	Cox proportional hazards regression
NHIS	National Health Insurance Service
AUC	Area under the ROC curve
