# Supplementary material for: Sex-Stratified Prediction Models for 5-Year Nonalcoholic Fatty Liver Disease Risk in Thyroid Cancer Patients: A Nationwide Cohort Study
Source: Biomedicines. 2025 Sep 12;13(9):2250. doi: 10.3390/biomedicines13092250 (PMC12467750; doi:10.3390/biomedicines13092250)
Supplement: Supplementary file 1 [file biomedicines-13-02250-s001.zip › biomedicines-3854901-supplementary.pdf]

Supplemental Table S1. Definitions of variables and outcome.

Diagnosis	ICD-10 code / Claim code	Diagnostic definition
**Outcome**		
NAFLD	K760, K758	
**Variables 1**		
Income	1) Low	1) 0,1,2,3 in decile
	2) Middle	2) 4,5,6,7 in decile
	3) High	3) 8,9,10 in decile
Residence	1) Urban	1) Seoul, Busan, Daegu, Incheon, Gwangju, Daejeon, and Ulsan
	2) Rural	2) Gyeonggi Province, Gangwon Province, North and South Chungcheong Provinces, North and South Jeolla
Alcohol intake		Number of alcohol consumption per week
Alcohol binge		Male: 5 drinks per session
		Female: 4 drinks per session
Regular exercise		Physical activity at least 3 times per week
Body mass index	1) Underweight	1) < 18.5 kg/m^2
	2) Normal	2) 18.5-22.9 kg/m^2
	3) Overweight	3) 23.0-24.9 kg/m^2
	4) Obese	4) ≥ 25 kg/m^2

Steroid use	193302ATB, 193305ATB, 217034ASY, 217001ATB, 193601BIJ, 193603BIJ, 193604BIJ, 217131COS, 217132COS, 217135COS, 217136COS, 217137COS, 217138COS, 217139COS, 193432COM, 193433COM, 193434CLT, 193435CLT, 193438CLT, 217531CCM, 217532CCM, 217533CCM, 217534CCM, 217535CLT, 217536CCM, 217537CCM, 141901ATB, 141903ATB, 142230BIJ,142232BIJ, 141930COO, 141931COS, 141906CIM, 142231COO, 141910CSI, 142130CCM, 142131CCM, 142133CCM, 243201ATB,243202ATB, 243203ATB, 243336BIJ, 243335BIJ, 243337BIJ, 243340COS, 243330CLQ, 243331CCM, 243339COM, 160201ATB, 170901ATB, 170906ATB, 170931COM, 170932CCM, 170947COM, 170938CLT, 170939CLT, 170942CLT, 170943CLT, 170933CSS, 170950CLT, 170941CLT, 170944CLT, 170945CLT, 170946CLT, 170934CSS, 171030CCM, 171130CCM	
Thyroidectomy	1) Lobectomy: P4551, P4553, P4554	
	2) Total thyroidectomy: P4561, P4552	
**Variables 2 (Comorbidities)**		
Hypertension	I10, I11, I12, I13, I15	Blood pressure ≥140/90 mmHg
Diabetes mellitus	E10–E14	Fasting plasma glucose ≥126 mg/dL
Dyslipidemia	E78	Total cholesterol ≥240 mg/dL
Obesity	E66	Body mass index ≥ 25 kg/m^2
**Variables 3 (Charlson Comorbidity Index, Score)**		
Myocardial infarction (1 point)	I21, I22, I252	
Congestive heart failure (1 point)	I099, I110, I130, I132, I255, I420, I425, I426, I427, I428, I429, I43, I50, P290	
Peripheral vascular disease	I70, I71, I731, I738, I739, I771, I790, I792, K551, K558, K559, Z958, Z959	

(1 point)	
Cerebrovascular disease (1 point)	G45, G46, I60, I61, I62, I63, I64, I65, I66, I67, I68, I69, H340
Dementia (1 point)	F00, F01, F02, F03, G30, F051, G311
Chronic pulmonary disease (1 point)	I278, I279, J40, J41, J42, J43, J44, J45, J46, J47, J60, J61, J62, J63, J64, J65, J66, J67, J684, J701, J703
Connective tissue disease (1 point)	M05, M06, M315, M32, M33, M34, M351, M353, M360
Peptic ulcer disease (1 point)	K25, K26, K27, K28
Mild liver disease (1 point)	B18, K700, K701, K702, K703, K709, K713, K714, K715, K717, K73, K74, K760, K762, K763, K764, K768, K769, Z944
Moderate or severe liver disease (3 points)	I850, I859, I864, I982, K704, K711, K721, K729, K765, K766, K767
Diabetes without complications (1 point)	E100, E101, E106, E108, E109, E110, E111, E116, E118, E119, E120, E121, E126, E128, E129, E130, E131, E136, E138, E139, E140, E141, E146, E148, E149
Diabetes with complications (2 points)	E102, E103, E104, E105, E107, E112, E113, E114, E115, E117, E122, E123, E124, E125, E127, E132, E133, E134, E135, E137, E142, E143, E144, E145, E147
Paraplegia and hemiplegia	G041, G114, G800, G801, G802, G81, G82, G830, G831, G832, G833, G834, G839

(2 points)	
Renal disease (2 points)	I120, I131, N030, N031, N032, N033, N034, N035, N036, N037, N038, N039, N050, N051, N052, N053, N054, N055, N056, N057, N058, N059, N18, N19, N250, Z490, Z491, Z492, Z940, Z992
Cancer (2 points)	C00, C01, C02, C03, C04, C05, C06, C07, C08, C09, C10, C11, C12, C13, C14, C15, C16, C17, C18, C19, C20, C21, C22, C23, C24, C25, C26, C30, C31, C32, C33, C34, C37, C38, C39, C40, C41, C43, C45, C46, C47, C48, C49, C50, C51, C52, C53, C54, C55, C56, C57, C58, C60, C61, C62, C63, C64, C65, C66, C67, C68, C69, C70, C71, C72, C73, C74, C75, C76, C81, C82, C83, C84, C85, C88, C90, C91, C92, C93, C94, C95, C96, C97
Metastatic carcinoma (6 points)	C77, C78, C79, C80
AIDS/HIV (6 points)	B20, B21, B22, B24

Variables retrieved from qualification and health examination data to assess demographic characteristics at the index date. ICD-10, International classification of disease 10th revision; AIDS, acquired immunodeficiency syndrome; COPD, chronic obstructive pulmonary disease; HIV, human immunodeficiency virus.

Supplemental Table S2. Optimal hyperparameters of random survival forest.

Male	Female		
	All	≤50 years	>50 years
mtry = 9	mtry = 5	mtry = 5	mtry = 9
ntree = 50	ntree = 30	ntree = 50	ntree = 30
node size = 15	node size = 15	node size = 3	node size = 10

Supplemental Table S3. Baseline characteristics of female populations.

Variables	≤50 years (N=1,576)	>50 years (N=1,433)	p value
Age	43.0 (36.0-47.0)	57.0 (53.0-63.0)	<.001
Income			0.011
Low	478 (30.3)	373 (26.0)	
Middle	437 (27.7)	389 (27.1)	
High	661 (41.9)	671 (46.8)	
Residence			0.434
Urban	800 (50.8)	706 (49.3)	
Rural	776 (49.2)	727 (50.7)	
Disability	17 (1.1)	86 (6.0)	<.001
Insurance type			<.001
Self-employed	398 (25.3)	467 (32.6)	

Work-employed	1,178 (74.7)	966 (67.4)	
Smoking status			0.055
Never	1,516 (96.2)	1,400 (97.7)	
Ex	25 (1.6)	15 (1.0)	
Current	35 (2.2)	18 (1.3)	
Alcohol intake			<.001
0	1,208 (76.6)	1,279 (89.3)	
1	323 (20.5)	136 (9.5)	
2	38 (2.4)	9 (0.6)	
≥ 3	7 (0.4)	9 (0.6)	
Alcohol binge	579 (36.7)	410 (28.6)	<.001
Regular exercise	185 (11.7)	222 (15.5)	0.003
**Health examination**			
BMI (kg/m^2)	22.3 (20.4-24.5)	24.0 (22.1-26.1)	<.001
BMI			<.001
Underweight	97 (6.2)	22 (1.5)	
Normal	821 (52.1)	489 (34.1)	
Overweight	324 (20.6)	377 (26.3)	
Obese	334 (21.2)	545 (38.0)	
SBP (mmHg)	114 (106-124)	124 (113-135)	<.001
DBP (mmHg)	70 (66-80)	78 (70-83)	<.001
FPG (mg/dL)	90 (84-97)	94 (86-104)	<.001
TC (mg/dL)	183 (163-207)	201 (177-226)	<.001
AST (IU/L)	19 (16-23)	23 (19-28)	<.001
ALT (IU/L)	15 (12-20)	20 (15-26)	<.001
GGT (IU/L)	15 (12-21)	18 (14-26)	<.001

**Comorbidities**			
Dyslipidemia	538 (34.1)	921 (64.3)	<.001
Diabetes	235 (14.9)	540 (37.7)	<.001
Hypertension	319 (20.2)	844 (58.9)	<.001
Obesity	496 (31.5)	897 (62.6)	<.001
CCI			<.001
≤2	986 62.6)	619 (43.2)	
>2	590 (37.4)	814 (56.8)	
**Thyroidectomy type**			0.051
Lobectomy	306 (19.4)	238 (16.6)	
Total thyroidectomy	1,270 (80.6)	1,195 (83.4)	
**Outcome**			
NAFLD	124 (7.9)	183 (12.8)	<.001

Values are expressed as mean (standard deviation) or number (%). BMI, body mass index; SBP, systolic blood pressure; DBP, diastolic blood pressure; FPG, fasting plasma glucose; TC, total cholesterol; ALT, alanine aminotransferase; AST, aspartate aminotransferase; GGT, gamma-glutamyl transferase; CCI, Charlson Comorbidity Score; NAFLD, nonalcoholic fatty liver disease

Supplemental Table S4. Baseline characteristics of the training and test set in male cohort.

Variables	Training set (N=440)	Test set (N=195)	p value
Age	47.0 (39.0-55.0)	49.0 (39.0-56.5)	0.360
Income			0.152
Low	48 (10.9)	32 (16.4)	
Middle	102 (23.2)	44 (22.6)	
High	290 (65.9)	119 (61.0)	

Residence			0.296
Urban	229 (52.0)	92 (47.2)	
Rural	211 (48.0)	103 (52.8)	
Disability	16 (3.6)	12 (6.2)	0.224
Insurance type			0.787
Self-employed	80 (18.2)	33 (16.9)	
Work-employed	360 (81.8)	162 (83.1)	
Smoking status			0.127
Never	182 (41.4)	66 (33.8)	
Ex	129 (29.3)	71 (36.4)	
Current	129 (29.3)	58 (29.7)	
Alcohol intake			0.558
0	184 (41.8)	84 (43.1)	
1	177 (40.2)	79 (40.5)	
2	63 (14.3)	29 (14.9)	
≥ 3	16 (3.6)	3 (1.5)	
Alcohol binge	245 (55.7)	108 (55.4)	1.000
Regular exercise	61 (13.9)	22 (11.3)	0.446
**Health examination**			
BMI (kg/m^2)	24.7 (23.0-26.6)	25.0 (23.2-27.0)	0.530
BMI			0.452
Underweight	5 (1.1)	4 (2.1)	
Normal	104 (23.6)	41 (21.0)	
Overweight	131 (29.8)	51 (26.2)	
Obese	200 (45.5)	99 (50.8)	
SBP (mmHg)	124 (116-130)	125 (117-132)	0.350

DBP (mmHg)	80 (70-85)	80 (71-84)	0.761
FPG (mg/dL)	94 (87-104.5)	96 (87-107)	0.212
TC (mg/dL)	193 (171-220)	189 (164.5-217)	0.426
AST (IU/L)	24 (20-29)	24 (20-30)	0.706
ALT (IU/L)	25 (18-35)	26 (20-35.5)	0.352
GGT (IU/L)	32.5 (23-49)	31 (21-45)	0.152
**Comorbidities**			
Dyslipidemia	221 (50.2)	104 (53.3)	0.525
Diabetes	119 (27.0)	55 (28.2)	0.837
Hypertension	246 (55.9)	106 (54.4)	0.783
Obesity	281 (63.9)	128 (65.6)	0.733
CCI			0.227
≤2	250 (56.8)	100 (51.3)	
>2	190 (43.2)	95 (48.7)	
**Thyroidectomy type**			0.183
Lobectomy	89 (20.2)	30 (15.4)	
Total thyroidectomy	351 (79.8)	165 (84.6)	
**Outcome**			
NAFLD	43 (9.8)	21 (10.8)	0.809

Values are expressed as mean (standard deviation) or number (%). BMI, body mass index; SBP, systolic blood pressure; DBP, diastolic blood pressure; FPG, fasting plasma glucose; TC, total cholesterol; ALT, alanine aminotransferase; AST, aspartate aminotransferase; GGT, gamma-glutamyl transferase; CCI, Charlson Comorbidity Score; NAFLD, nonalcoholic fatty liver disease

Supplemental Table S5. Baseline characteristics of the training and test set in female cohort.

Variables	Training set (N=2,103)	Test set (N=906)	p value
Age	50.0 (42.0-57.0)	50.0 (43.0-56.0)	0.595
Income			0.634
Low	601 (28.6)	250 (27.6)	
Middle	583 (27.7)	243 (26.8)	
High	919 (43.7)	413 (45.6)	
Residence			0.688
Urban	1,047 (49.8)	459 (50.7)	
Rural	1,056 (50.2)	447 (49.3)	
Disability	66 (3.1)	37 (4.1)	0.230
Insurance type			0.864
Self-employed	607 (28.9)	258 (28.5)	
Work-employed	1,496 (71.1)	648 (71.5)	
Smoking status			0.516
Never	2,033 (96.7)	883 (97.5)	
Ex	30 (1.4)	10 (1.1)	
Current	40 (1.9)	13 (1.4)	
Alcohol intake			0.324
0	1,750 (83.2)	737 (81.3)	
1	313 (14.9)	146 (16.1)	
2	28 (1.3)	19 (2.1)	
≥ 3	12 (0.6)	4 (0.4)	
Alcohol binge	693 (33.0)	296 (32.7)	0.913
Regular exercise	295 (14.0)	112 (12.4)	0.243

**Health examination**			
BMI (kg/m^2)	23.1 (21.2-25.3)	23.1 (21.2-25.2)	0.842
BMI			0.849
Underweight	85 (4.0)	34 (3.8)	
Normal	915 (43.5)	395 (43.6)	
Overweight	482 (22.9)	219 (24.2)	
Obese	621 (29.5)	258 (28.5)	
SBP (mmHg)	120 (110-130)	120 (110-130)	0.799
DBP (mmHg)	74 (69-80)	74 (69-80)	0.901
FPG (mg/dL)	92 (85-100)	92 (85-99)	0.995
TC (mg/dL)	192 (168-218)	190 (168-218)	0.640
AST (IU/L)	21 (17-25)	21 (17-25)	0.993
ALT (IU/L)	17 (13-23)	18 (13-24)	0.593
GGT (IU/L)	16 (12-23)	17 (13-24)	0.135
**Comorbidities**			
Dyslipidemia	1025 (48.7)	434 (47.9)	0.703
Diabetes	542 (25.8)	233 (25.7)	1.000
Hypertension	813 (38.7)	350 (38.6)	1.000
Obesity	996 (47.4)	397 (43.8)	0.081
CCI			0.196
≤2	1,105 (52.5)	500 (55.2)	
>2	998 (47.5)	406 (44.8)	
**Thyroidectomy type**			0.129
Lobectomy	365 (17.4)	179 (19.8)	
Total thyroidectomy	1,738 (82.6)	727 (80.2)	
**Outcome**			

NAFLD	214 (10.2)	93 (10.3)	0.993

Values are expressed as mean (standard deviation) or number (%). BMI, body mass index; SBP, systolic blood pressure; DBP, diastolic blood pressure; FPG, fasting plasma glucose; TC, total cholesterol; ALT, alanine aminotransferase; AST, aspartate aminotransferase; GGT, gamma- glutamyl transferase; CCI, Charlson Comorbidity Score; NAFLD, nonalcoholic fatty liver disease

Supplemental Table S6. Baseline characteristics of the training and test set in younger (≤50 years) female cohort.

Variables	Training set (N=1,099)	Test set (N=477)	p value
Age	43.0 (36.0-47.0)	43.0 (37.0-47.0)	0.948
Income			0.215
Low	348 (31.7)	130 (27.3)	
Middle	298 (27.1)	139 (29.1)	
High	453 (41.2)	208 (43.6)	
Residence			0.632
Urban	553 (50.3)	247 (51.8)	
Rural	546 (49.7)	230 (48.2)	
Disability	9 (0.8)	8 (1.7)	0.211
Insurance type			0.805
Self-employed	280 (25.5)	118 (24.7)	
Work-employed	819 (74.5)	359 (75.3)	
Smoking status			0.129
Never	1,063 (96.7)	453 (95.0)	
Ex	13 (1.2)	12 (2.5)	
Current	23 (2.1)	12 (2.5)	

Alcohol intake			0.768
0	842 (76.6)	366 (76.7)	
1	226 (20.6)	97 (20.3)	
2	25 (2.3)	13 (2.7)	
≥ 3	6 (0.5)	1 (0.2)	
Alcohol binge	392 (35.7)	187 (39.2)	0.200
Regular exercise	126 (11.5)	59 (12.4)	0.669
**Health examination**			
BMI (kg/m^2)	22.3 (20.4-24.4)	22.3 (20.3-24.7)	0.805
BMI			0.676
Underweight	64 (5.8)	33 (6.9)	
Normal	580 (52.8)	241 (50.5)	
Overweight	228 (20.7)	96 (20.1)	
Obese	227 (20.7)	107 (22.4)	
SBP (mmHg)	114 (106-124)	113 (106-124)	0.976
DBP (mmHg)	70 (66-80)	70 (65-80)	0.402
FPG (mg/dL)	90 (84-97)	90 (83-97)	0.719
TC (mg/dL)	184 (162-207)	181 (163-205)	0.577
AST (IU/L)	19 (16-23)	19 (16-22)	0.371
ALT (IU/L)	15 (12-20)	15 (12-20)	0.882
GGT (IU/L)	15 (11-20)	15 (12-21)	0.364
**Comorbidities**			
Dyslipidemia	375 (34.1)	163 (34.2)	1.000
Diabetes	163 (14.8)	72 (15.1)	0.954
Hypertension	220 (20.0)	99 (20.8)	0.79
Obesity	336 (30.6)	160 (33.5)	0.268

CCI			0.993
≤2	687 (62.5)	299 (62.7)	
>2	412 (37.5)	178 (37.3)	
**Thyroidectomy type**			1.000
Lobectomy	213 (19.4)	93 (19.5)	
Total thyroidectomy	886 (80.6)	384 (80.5)	
**Outcome**			
NAFLD	85 (7.7)	39 (8.2)	0.843

Values are expressed as mean (standard deviation) or number (%). BMI, body mass index; SBP, systolic blood pressure; DBP, diastolic blood pressure; FPG, fasting plasma glucose; TC, total cholesterol; ALT, alanine aminotransferase; AST, aspartate aminotransferase; GGT, gamma-glutamyl transferase; CCI, Charlson Comorbidity Score; NAFLD, nonalcoholic fatty liver disease

Supplemental Table S7. Baseline characteristics of the training and test set in older (>50 years) female cohort.

Variables	Training set (N=1,001)	Test set (N=432)	p value
Age	57.0 (53.0-63.0)	58.0 (53.0-63.0)	0.929
Income			0.911
Low	262 (26.2)	111 (25.7)	
Middle	274 (27.4)	115 (26.6)	
High	465 (46.5)	206 (47.7)	
Residence			0.266
Urban	483 (48.3)	223 (51.6)	
Rural	518 (51.7)	209 (48.4)	
Disability	53 (5.3)	33 (7.6)	0.111
Insurance type			0.599

Self-employed	331 (33.1)	136 (31.5)	
Work-employed	670 (66.9)	296 (68.5)	
Smoking status			0.663
Never	977 (97.6)	423 (97.9)	
Ex	12 (1.2)	3 (0.7)	
Current	12 (1.2)	6 (1.4)	
Alcohol intake			0.868
0	898 (89.7)	381 (88.2)	
1	91 (9.1)	45 (10.4)	
2	6 (0.6)	3 (0.7)	
≥ 3	6 (0.6)	3 (0.7)	
Alcohol binge	283 (28.3)	127 (29.4)	0.712
Regular exercise	162 (16.2)	60 (13.9)	0.307
**Health examination**			
BMI (kg/m^2)	24.0 (22.2-26.1)	23.9 (22.1-26.3)	0.816
BMI			0.219
Underweight	11 (1.1)	11 (2.5)	
Normal	340 (34.0)	149 (34.5)	
Overweight	268 (26.8)	109 (25.2)	
Obese	382 (38.2)	163 (37.7)	
SBP (mmHg)	124 (113-135)	124 (113.5-134)	0.951
DBP (mmHg)	78 (70-83)	77 (70-83)	0.647
FPG (mg/dL)	94 (86-103)	94 (86.5-104.5)	0.919
TC (mg/dL)	200 (177-226)	202 (176-226.5)	0.749
AST (IU/L)	23 (19-27)	23 (19-28)	0.996
ALT (IU/L)	20 (15-26)	20 (15-26)	0.403

GGT (IU/L)	18 (14-27)	18 (14-25)	0.826
**Comorbidities**			
Dyslipidemia	649 (64.8)	272 (63.0)	0.536
Diabetes	372 (37.2)	168 (38.9)	0.576
Hypertension	593 (59.2)	251 (58.1)	0.731
Obesity	637 (63.6)	260 (60.2)	0.238
CCI			0.807
≤2	435 (43.5)	184 (42.6)	
>2	566 (56.5)	248 (57.4)	
**Thyroidectomy type**			0.058
Lobectomy	179 (17.9)	59 (13.7)	
Total thyroidectomy	822 (82.1)	373 (86.3)	
**Outcome**			
NAFLD	126 (12.6)	57 (13.2)	0.818

Values are expressed as mean (standard deviation) or number (%). BMI, body mass index; SBP, systolic blood pressure; DBP, diastolic blood pressure; FPG, fasting plasma glucose; TC, total cholesterol; ALT, alanine aminotransferase; AST, aspartate aminotransferase; GGT, gamma-glutamyl transferase; CCI, Charlson Comorbidity Score; NAFLD, nonalcoholic fatty liver disease

Supplemental Table S8. C-index of prediction models in training set.

	RSF		Cox	
	C-index (95% CI)	p value	C-index (95% CI)	p value
**Male**	0.92 (0.89-0.95)	<.001	0.62 (0.54-0.70)	0.450
**Female**	0.97 (0.96-0.98)	<.001	0.67 (0.63-0.70)	<.001
≤50 years	0.93 (0.91-0.95)	<.001	0.69 (0.63-0.74)	<.001

>50 years	0.95 (0.94-0.96)	<.001	0.64 (0.59-0.68)	<.001

RSF, random survival forest; Cox, Cox proportional hazards regression; C-index, concordance index; CI, confidence interval.

Supplemental Table S9. Hazard ratio of the female-specific RSF model in test set.

		Total	Event (%)	1,000PY	HR (95% CI)	p value
All female	Low risk	796	76 (9.55)	7349.46	1.00	
	High risk	110	17 (15.45)	12299.81	1.68 (0.99-2.84)	0.054
≤50 years	Low risk	435	30 (6.90)	5240.02	1.00	
	High risk	42	9 (21.43)	17833.61	3.4 (1.61-7.16)	0.001
>50 years	Low risk	386	49 (12.69)	9977.94	1.00	
	High risk	46	8 (17.39)	14187.4	1.42 (0.67-3.00)	0.355

RSF, random survival forest; 1,000PY, per 1000 person-year; HR, hazard ratio; CI, confidence interval.

Supplemental Table S10. Hazard ratio of the male-specific models in test set.

		Total	Event (%)	1,000PY	HR (95% CI)	p value
RSF	Low risk	174	18 (10.34)	8004.97	1.00	
	High risk	21	3 (14.29)	11433.01	1.42 (0.42-4.83)	0.573
Cox	Low risk	113	11 (9.73)	7439.11	1.00	
	High risk	82	10 (12.20)	9667.09	1.3 (0.55-3.05)	0.554

RSF, random survival forest; Cox, Cox proportional hazards regression; 1,000PY, per 1000 person-year; HR, hazard ratio; CI, confidence interval.

Supplemental Table S11. Hazard ratio of the female-specific Cox model in training set.

		Total	Event (%)	1,000PY	HR (95% CI)	p value
All female	Low risk	1518	99 (6.52)	4940.85	1.00	
	High risk	585	115 (19.66)	15947.43	3.24 (2.48-4.24)	<.001
≤50 years	Low risk	714	31 (4.34)	3246.69	1.00	
	High risk	385	54 (14.03)	11043.15	3.42 (2.20-5.31)	<.001
>50 years	Low risk	764	71 (9.29)	7129.95	1.00	
	High risk	237	55 (23.21)	19287.54	2.72 (1.91-3.87)	<.001

Cox, Cox proportional hazards regression; 1,000PY, per 1000 person-year; HR, hazard ratio; CI, confidence interval.

Supplemental Table S12. Hazard ratio of the female-specific RSF model in training set.

		Total	Event (%)	1,000PY	HR (95% CI)	p value
All female	Low risk	1876	37 (1.97)	1451	1.00	
	High risk	227	177 (77.97)	102342.4	85 (59.29-121.86)	<.001
≤50 years	Low risk	985	22 (2.23)	1650.69	1.00	
	High risk	114	63 (55.26)	56739.78	36.76 (22.57-59.90)	<.001
>50 years	Low risk	897	41 (4.57)	3390.04	1.00	
	High risk	104	85 (81.73)	118827.3	43.26 (29.49-63.48)	<.001

RSF, random survival forest; 1,000PY, per 1000 person-year; HR, hazard ratio; CI, confidence interval.

Supplemental Table S13. Hazard ratio of the male-specific models in training set.

		Total	Event (%)	1,000PY	HR (95% CI)	p value
RSF	Low risk	394	17 (4.31)	3206.74	1.00	
	High risk	46	26 (56.52)	66695.87	21.64 (11.68-40.11)	<.001
Cox	Low risk	268	13 (4.85)	3632.47	1.00	
	High risk	172	30 (17.44)	14216.53	3.92 (2.04-7.51)	<.001

RSF, random survival forest; Cox, Cox proportional hazards regression; 1,000PY, per 1000 person-year; HR, hazard ratio; CI, confidence interval.

<<Supplementary Figure>>

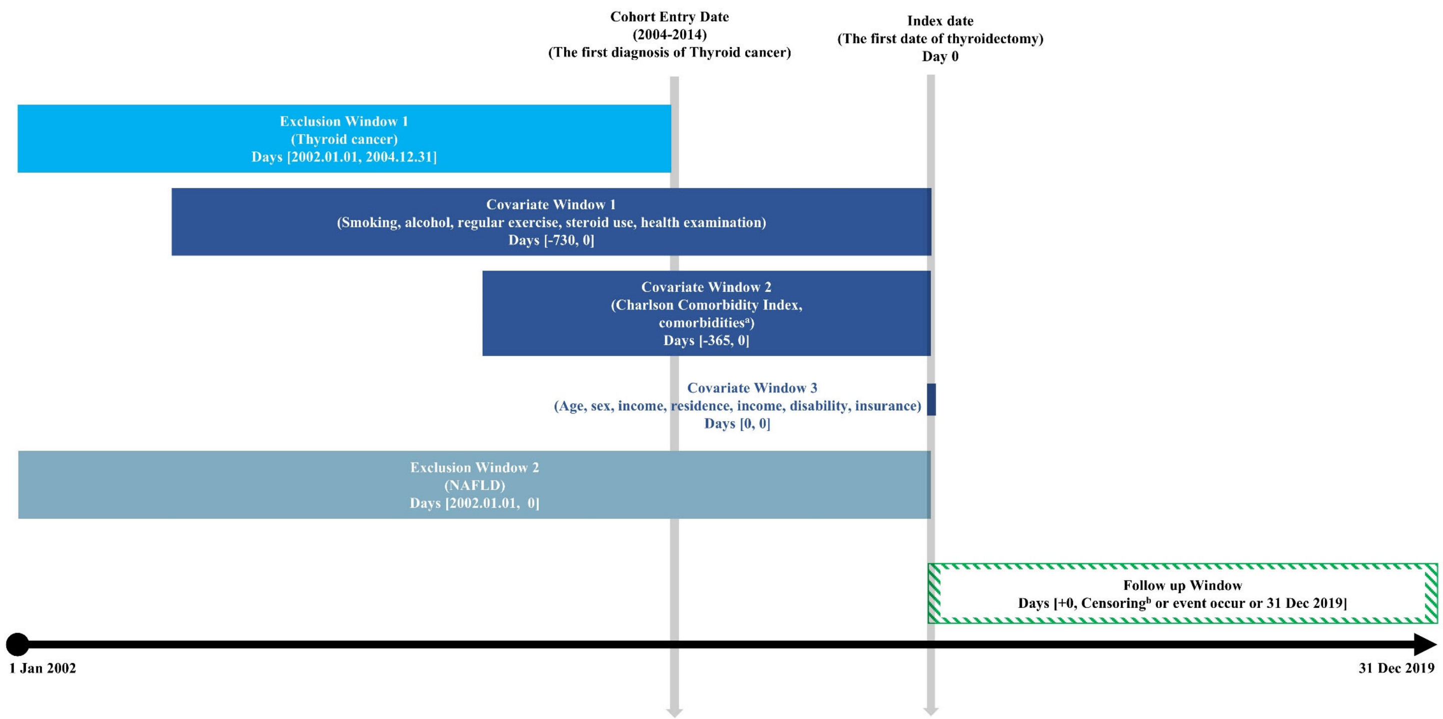

Supplemental Figure S1. Study design. Patients were followed-up from the index date until incidence of NAFLD, death, or December 31, 2019. [a]Comorbidities include hypertension, diabetes, dyslipidemia, and obesity. [b]Follow-up was censored at the earliest occurrence of death or 5 years from the index date. NAFLD, nonalcoholic fatty liver disease.

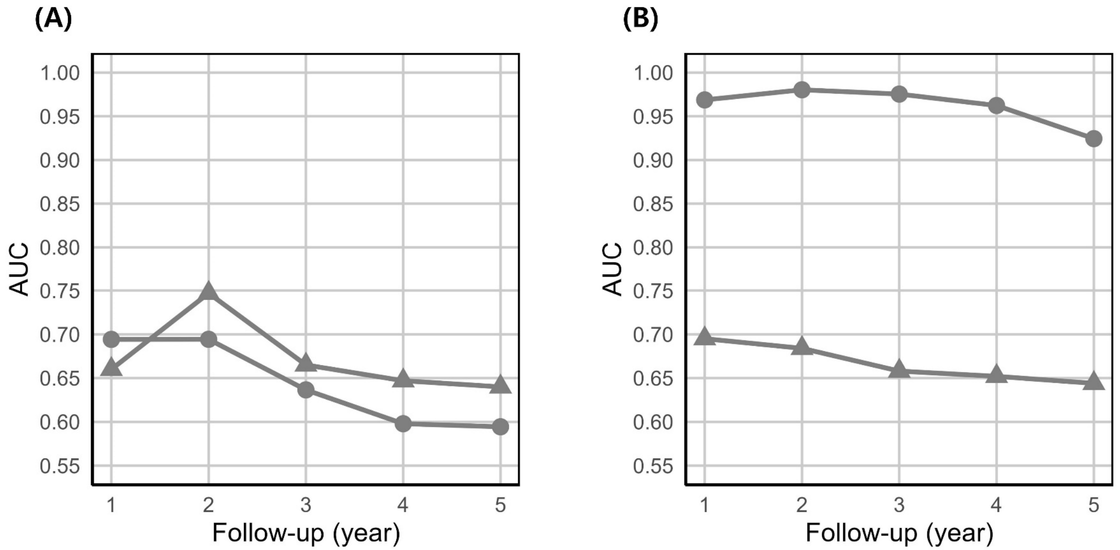

Supplemental Figure S2. Time-dependent AUC of male-specific models. (A) test set (B) training set. ▲, Cox proportional hazards regression (Cox) model. ●, Random survival forest (RSF) model. AUC, area under the curve.

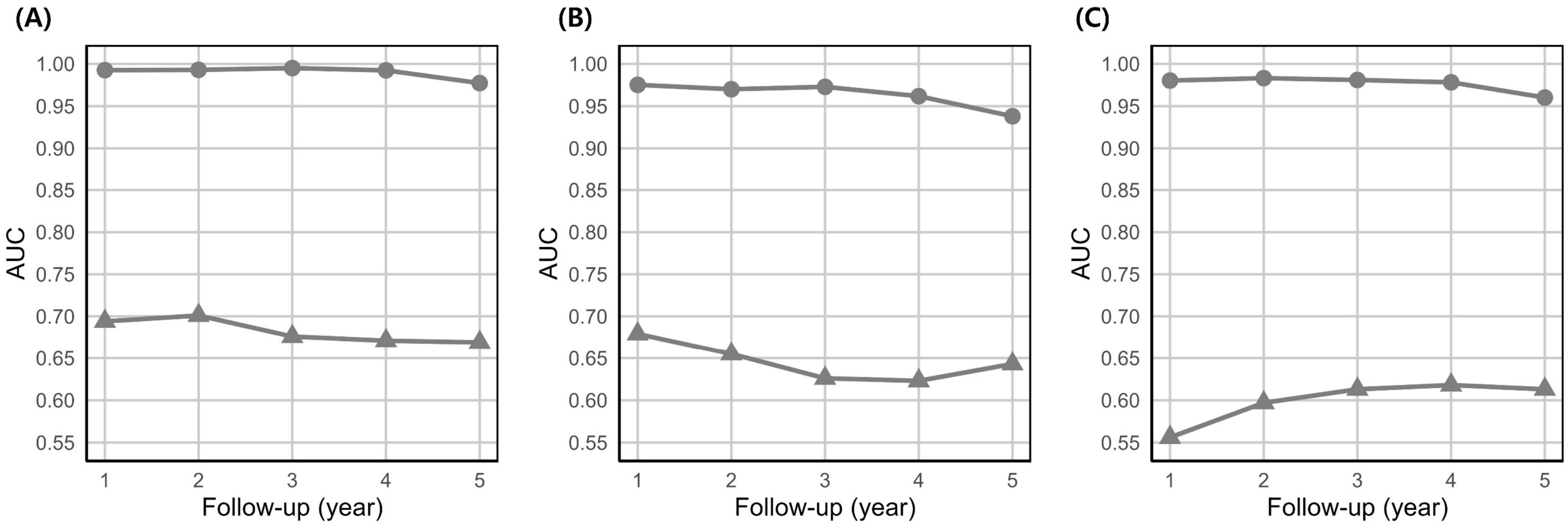

Supplemental Figure S3. Time-dependent AUC of female-specific models in training set. (A) all female (B) younger (≤50 years) female (C) older (>50 years) female. ▲, Cox proportional hazards regression (Cox) model. ●, Random survival forest (RSF) model. AUC, area under the curve.

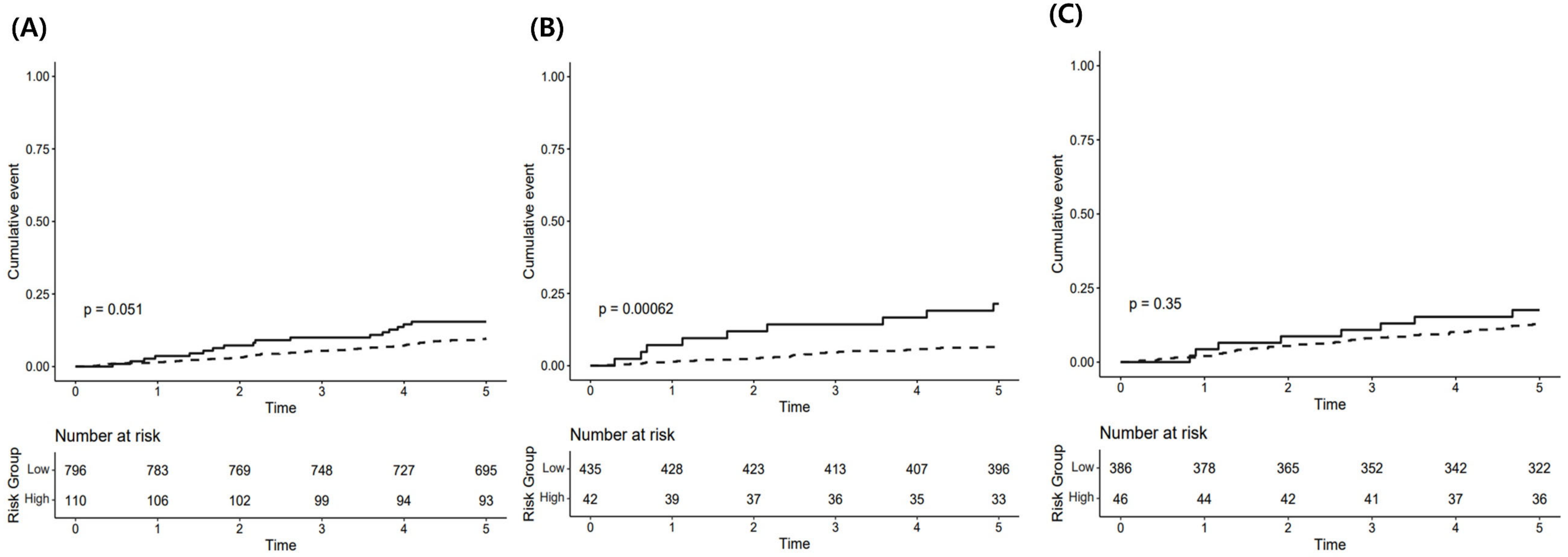

Supplemental Figure S4. Risk stratification of female-specific RSF models in test set. (A) all female (B) younger (≤50 years) female (C) older (>50 years) female. Solid line represents the high-risk group. Dash line represents the low-risk group.

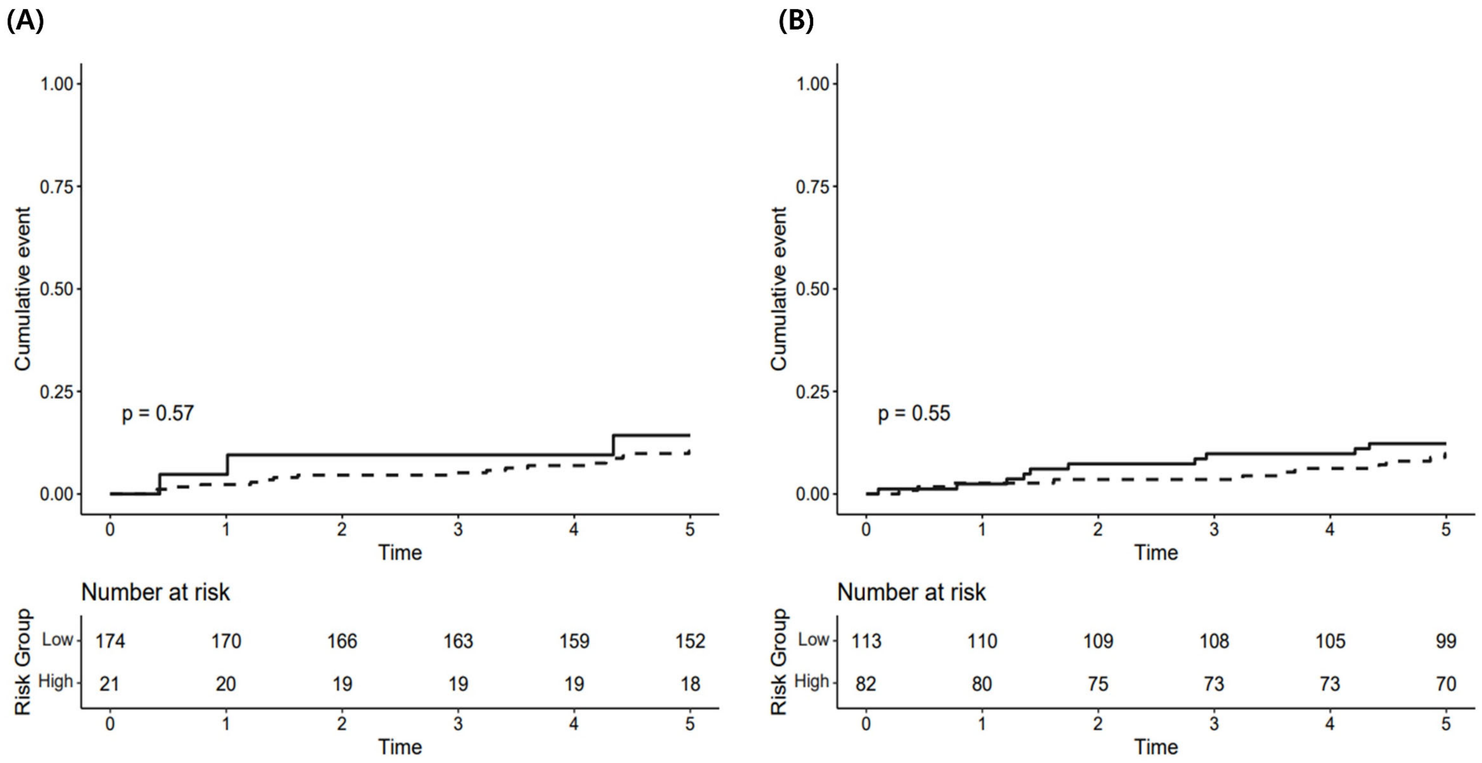

Supplemental Figure S5. Risk stratification of male-specific models in test set. (A) Random survival forest (RSF) model (B) Cox proportional hazards regression (Cox) model. Solid line represents the high-risk group. Dash line represents the low-risk group.

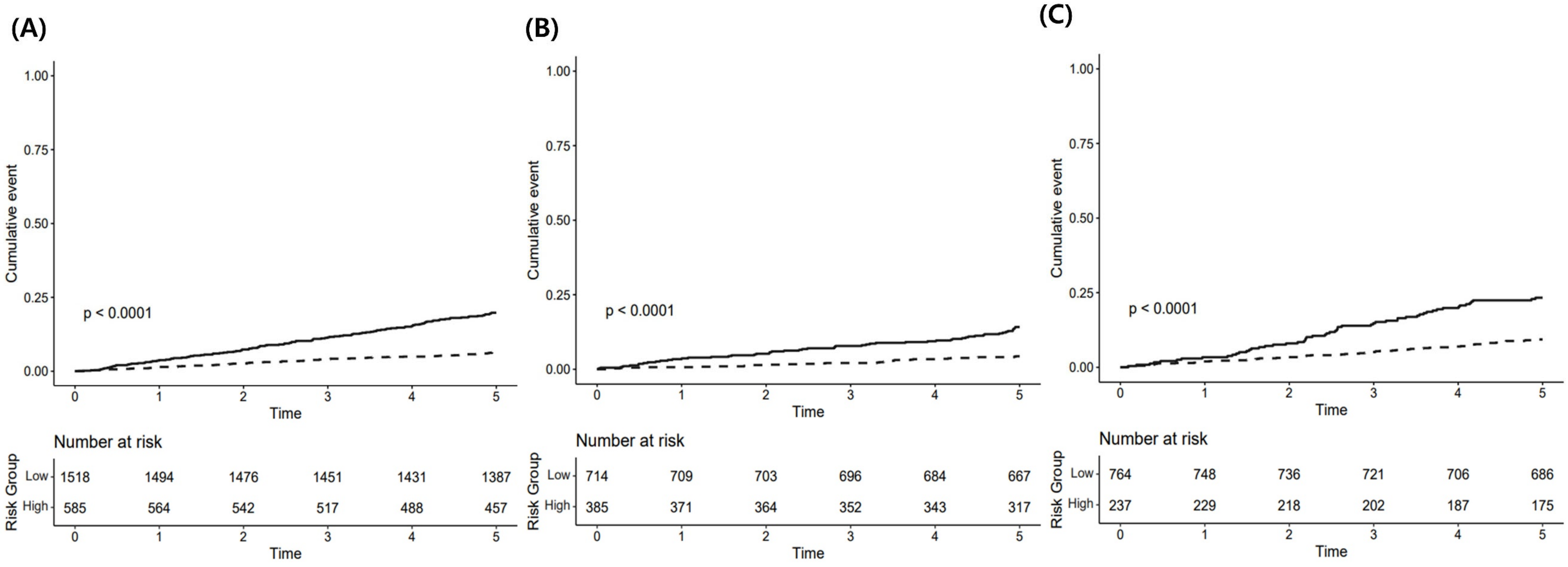

Supplemental Figure S6. Risk stratification of female-specific Cox models in training set. (A) all female (B) younger (≤50 years) female (C) older (>50 years) female. Solid line represents the high-risk group. Dash line represents the low-risk group.

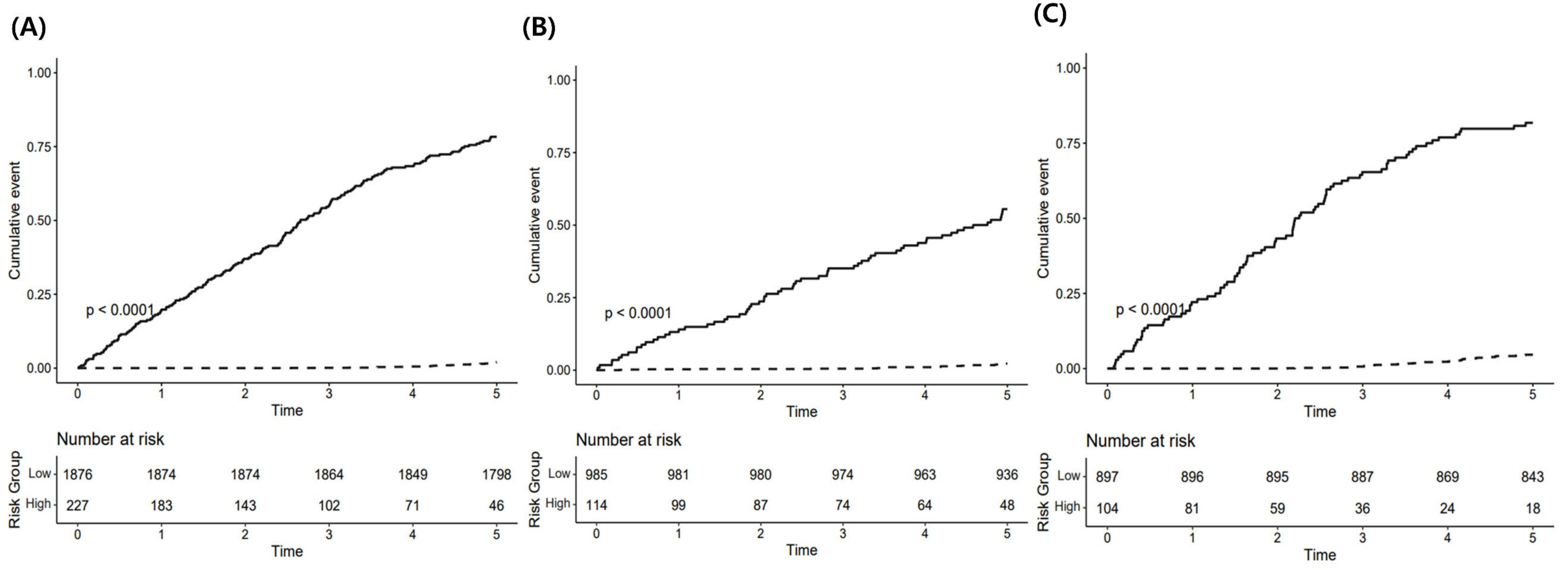

Supplemental Figure S7. Risk stratification of female-specific RSF models in training set. (A) all female (B) younger (≤50 years) female (C) older (>50 years) female. Solid line represents the high-risk group. Dash line represents the low-risk group.

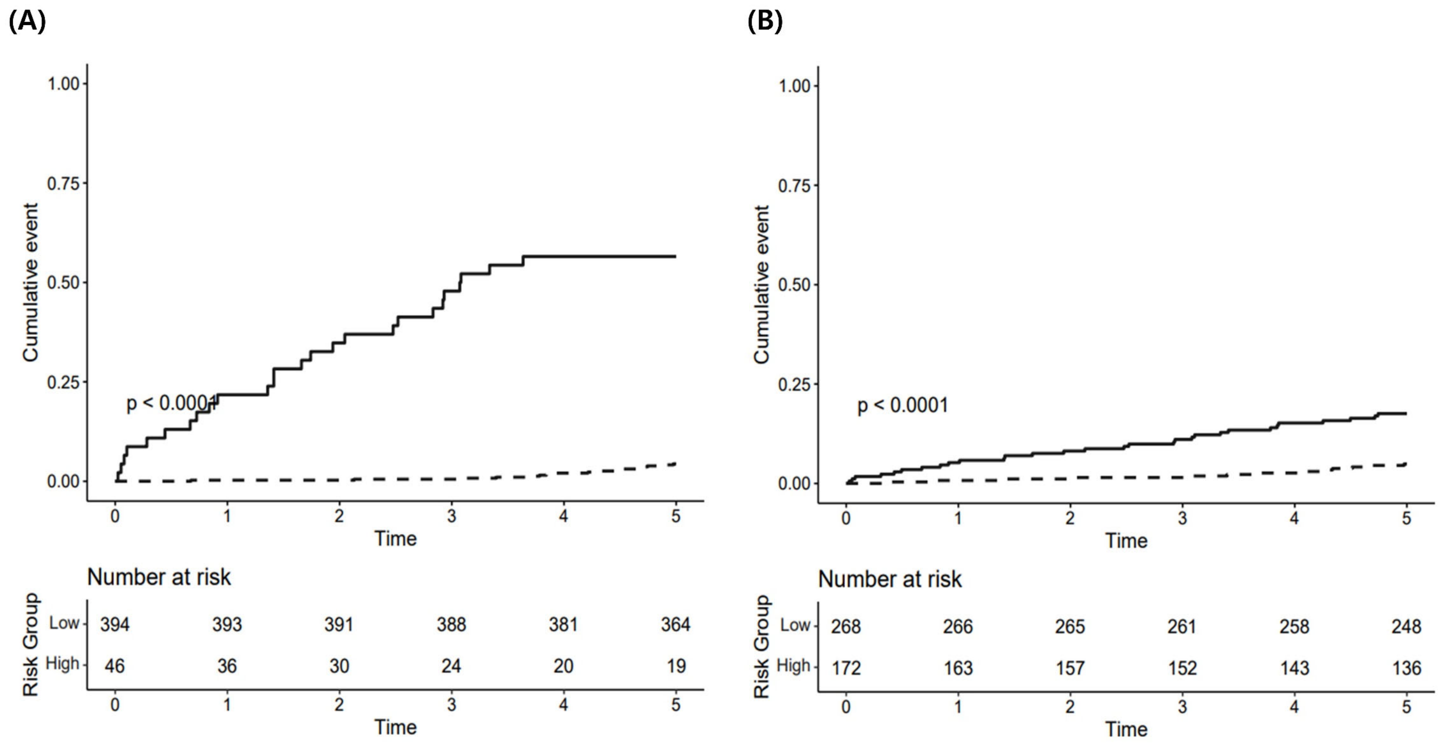

Supplemental Figure S8. Risk stratification of male-specific models in training set. (A) Random survival forest (RSF) model (B) Cox proportional hazards regression (Cox) model. Solid line represents the high-risk group. Dash line represents the low-risk group.
